# Berberine Suppressed Tumor Growth through Regulating Fatty Acid Metabolism and Triggering Cell Apoptosis via Targeting FABPs

**DOI:** 10.1155/2020/6195050

**Published:** 2020-04-08

**Authors:** Lingli Li, Ze Peng, Qian Hu, Lijun Xu, Xin Zou, Yan Yu, Dongmei Huang, Ping Yi

**Affiliations:** ^1^Institute of Integrated Traditional Chinese and Western Medicine, Tongji Hospital, Tongji Medical College, Huazhong University of Science and Technology, Wuhan, Hubei 430030, China; ^2^Department of Integrated Traditional Chinese and Western Medicine, West China Hospital of Sichuan University, Chengdu, Sichuan Province 610041, China; ^3^Wuhan Britain-China School, Wuhan, Hubei 430030, China; ^4^Department of Integrated Traditional Chinese and Western Medicine, Tongji Hospital, Tongji Medical College, Huazhong University of Science and Technology, Wuhan 430030, China

## Abstract

**Aim:**

To further investigate the mechanism behind the antitumor properties of berberine regarding lipid metabolism.

**Methods:**

Cell viability, proliferation, and apoptosis assays were performed to determine the antigrowth effects of berberine in vitro. Ectopic xenograft models in Balb/c nude mice were established to determine the antitumor effects of berberine in vivo.

**Results:**

Berberine inhibited cell viability and proliferation of MGC803 human gastric cancer cell lines in a time- and dose-dependent manner. Berberine induced apoptosis of MGC803 and increased the apoptotic rate with higher doses. Berberine induced the accumulation of fatty acid of MGC803 and suppressed the protein expression of FABPs and PPAR*α*. The FABP inhibitor BMS309403 recapitulated the effects of berberine on MGC803 cells. In the xenograft model, berberine significantly decreased the tumor volume and tumor weight and induced apoptosis in tumor tissues. Berberine significantly elevated the fatty acid content and inhibited the expression of FABPs and PPAR*α* in the MGC803 xenograft models.

**Conclusion:**

Berberine exerted anticancer effects on human gastric cancer both in vitro and in vivo by inducing apoptosis, which was due to the reduced protein expression of FABPs and the accumulation of fatty acid.

## 1. Introduction

An estimated 4.3 million new cancer cases and 2.9 million new cancer deaths occurred in China in 2018. China has lower cancer incidence but a 30% and 40% higher cancer mortality than the UK and USA, among which 36.4% of the cancer-related deaths were from the digestive tract cancers (stomach, liver, and esophagus cancer) and have relatively poorer prognoses [1]. Stomach cancer happened worldwide and is responsible for over 1,000,000 new cases in 2018 and an estimated 783,000 deaths (equating to 1 in every 12 deaths globally), making it the fifth most frequently diagnosed cancer and the third leading cause of cancer death [2]. The latest China cancer report in 2018 showed that gastric cancer ranks second in the incidence and mortality of cancer in China. One reason for the higher mortality in China may be the low-stage cancers at diagnosis and nonuniformed clinical cancer treatment strategies performed by different regions [1]. Therefore, it is important to determine a new specific target for gastric cancer and develop a newly adjuvant drug.

Lipids are widely distributed in cellular organelles and are critical components of all membranes. In addition to their role as structural components, lipids in membranes also serve important functions. Lipids not only function as second messengers to transduce signals within cells but also serve as important energy sources when nutrients are limited [3]. Accumulating experiments have demonstrated that lipid metabolism is substantially reprogrammed in cancers [4–6]. The obvious feature of lipid metabolism in cancers is an increased rate of lipogenesis and mitochondrial fatty acid *β*-oxidation. Gastric cancer demonstrates a similar tendency and displays typical changes regarding the varieties of metabolites involved in lipid metabolism [7], suggesting that the lipid metabolic pathway might be a potential pathway for gastric cancer therapy.

FABPs, members of the superfamily of lipid-binding proteins, are key lipid metabolic enzymes responsible for the regulation of fatty acid uptake and intracellular transport [8]. It was reported that FABPs were closely associated with cancer development and were upregulated in many cancers such as renal cell carcinoma [9], hepatocellular carcinoma [10], and breast cancer [11], which indicates the potential role of FABPs as the tumor markers and therapeutic targets in some cases.

Traditional Chinese medicine (TCM) has been practiced for thousands of years and now is widely accepted as an alternative treatment for cancers [12]. Berberine, extracted from Chinese herbs such as Coptis, is traditionally used to treat gastrointestinal infections such as bacterial diarrhea in China for a long time. Recent investigations have discovered its properties against many diseases including obesity, hypolipidemia [13], and gastric cancers [14]. Our previous study proved that berberine could attenuate the proliferation and induce cell cycle arrest of gastric cancer cell lines by targeting the AMPK/HNF4*α*/WNT5a pathway [15]. It was also confirmed that berberine inhibited the growth and induced apoptosis of gastric cancer cell lines, which effects were linked to inhibition of EGFR signals [14]. However, whether berberine exerted growth inhibition effects against tumors through regulating the lipid metabolism remained to be elucidated.

Therefore, we conducted this study to further investigate the mechanism behind the antitumor properties of berberine regarding lipid metabolism.

## 2. Materials and Methods

### 2.1. Materials and Reagents

Berberine chloride with a purity of more than 98%, isolated from Chinese herbal Huanglian, was purchased from Sigma-Aldrich, United States. Antibodies against FABP4, FABP5, and Bax were obtained from Cell Signaling Technologies (Boston, MA, United States). Rabbit antibodies against Bcl-2 and cleaved-caspase3 were purchased from ABclonal (China). Antibodies against GAPDH and *β*-actin were gained from Hubei Biossci Biological Co., Ltd. Rabbit antibodies against PPAR*α* and Bax were bought from proteintech Biotech Co., Ltd. Triglyceride detection kit and total cholesterol detection kit were obtained from Nanjing Jiancheng Bioengineering Institute. Annexin V-FITC/PI apoptosis detection kit was purchased from Biolegend Biotech Co., Ltd. The TUNEL detection kit was gained from Yeasen Biotech Co., Ltd.

### 2.2. Cell Culture

The gastric cancer cell line MGC803 was purchased from Wuhan Servicebio Technology Co., Ltd. MGC803 cells were routinely cultured in the RPMI-1640 medium (HyClone, China) supplemented with 10% fetal bovine serum (FBS) (SiJiQing, China) and 1% penicillin-streptomycin solution (Beijing Solarbio Science & Technology Co., Ltd.) at 37°C in a humidified atmosphere with 5% CO_2_.

### 2.3. MTT Assays

The cytotoxicity of berberine against MGC803 cells was determined with 3-(4,5-dimethyl-2-tetrazolyl)-2,5-diphenyl-2H tetrazolium bromide (MTT) assay. Cells with a number of 4000 cells per well were seeded in 96-well culture plates. After well adhered, the cells were starved with the RPMI-1640 medium supplemented with no FBS and 1% penicillin-streptomycin solution for 12 h and subsequently treated with different concentrations of berberine for 24 h, 48 h, or 72 h. Then, after removing the supernatant medium of each well, a volume of 100 *μ*l MTT solution with a concentration of 0.5 mg/ml was added to it, followed by incubation for 4 h at 37°C. The MTT solution was then removed, and another 150 *μ*l DMSO solution was added into each well. After shaking at a lower speed for 30 minutes to fully dissolve the crystals, the absorbance of each well at 570 nm was measured using an enzyme-linked immunoassay (Synergr2 BioTek, United States).

### 2.4. Apoptosis Assays

For apoptosis detection, MGC803 cells were dual stained using the Annexin V-FITC/PI apoptosis detection kit. Briefly, after treatment with berberine or inhibitor, cells were washed twice with PBS and trypsinized with trypsin solution without EDTA. Collected cells were then washed twice with cold PBS, resuspended in cold 1x binding buffer, and dual stained with Annexin V-FITC and PI in the dark at room temperature for 5 minutes. Flow cytometry was immediately performed on cells using the BD Accuri C6 flow cytometer (BD Biosciences) and analyzed using FlowJo v.10 software. Besides, TUNEL assays were also performed to detect the apoptosis induced by berberine in the tumor tissues in xenografts. All the TUNEL assays were conducted following the instructions of the TUNEL apoptosis detection kit(Alexa Fluor 488) provided by Yeasen Biotech Co., Ltd.

### 2.5. Measurement of Total Cholesterol and Triglyceride Content

The total cholesterol content and the triglyceride content of MGC803 cells and its supernatants and tumor tissues from MGC803 xenograft models were tested using total cholesterol content detection kit and the triglyceride content detection kit purchased from Nanjing Jiancheng Bioengineering Institute. Following the instructions, the supernatants, the serum sample, cell homogenate (10^7^ cells/200 *μ*l), and tumor tissue homogenate (10 mg/90 *μ*l) were added to 96-well plates with a volume of 2.5 *μ*l per well. And then the operation fluid provided by the detection kit with a volume of 250 *μ*l was added into per well above. The mixture was incubated at 37°C for 10 min. The absorbance of the wells was measured at 510 nm using the enzyme-linked immunoassay (Synergr2 BioTek, United States).

### 2.6. In Vivo Xenograft Models

Four-week-old female Balb/c nude mice weighed about 13–16 g were purchased from Hunan Slake Jingda Experimental Co., Ltd. (Hunan, China) and were maintained under specific pathogen-free atmosphere using a laminar airflow rack and had free access to sterilized food and autoclaved water. After a week of adjustable feeding, those mice were injected subcutaneously with MGC803 cells about 10^7^ cells per mouse into the right flank. Seventy-two hours later, a mass of more than 5 mm in maximal diameter in all mice was identifiable. Subsequently, the MGC803 xenograft mouse models were randomly assigned into two groups (control group and berberine group) followed by treatment with intragastric administration of normal saline (N group) or berberine (100 mg/kg, berberine group), respectively, for 18 days, and then all animals were sacrificed and the tumor tissues were abstracted and weighed. Furthermore, the weight and the tumor length and width were measured every three days. And the tumor volume were calculated with the following formula: tumor volume (mm³) = [tumor length (mm) *∗* tumor width (mm)^2^]/2. All animal experiments were performed according to the institutional guidelines and regulations. All animal studies were approved by the Huazhong University of Science and Technology Institutional Animal Care and Use Committee (S787).

### 2.7. Western Blot

The total proteins were extracted from the cultured MGC803 cells and the xenograft tumor tissues, respectively. The proteins were then separated by electrophoresis on a polyacrylamide gel and transferred to the nitrocellulose filter membrane (the NC membrane) (0.45 *μ*m or 0.22 *μ*m, Millipore, United States). After blocking with 5% no-fat milk (Wuhan Servicebio Technology Co., Ltd) for about 1 h, the membranes were incubated overnight at 4°C with primary antibodies, anti-FABP4 (1 : 1000), anti-FABP5 (1 : 1000), anticleaved caspased3 (1 : 1000), anti-Bcl-2 (1 : 1000), anti-Bax (1 : 1500), anti-PPAR*α* (1 : 1000), anti-GAPDH (1 : 10000), and anti-*β*-actin (1 : 5000). After having been washed three times with TBST solution, the membranes were incubated with the second antibody (anti-rabbit IgG (H + L) (DyLight™ 800 4x PEG Conjugate) #5151 for 1 : 30000 or anti-mouse IgG (H + L) (DyLight™ 800 4x PEG Conjugate) #5257 for 1 : 15000) at room temperature in the dark for 1 h and subsequently were scanned and visualized with a near-infrared double color laser imaging system (Odyssey, Lincoln, NE, United States).

### 2.8. Statistical Analysis

All experiments were conducted at least three times. All data were expressed as means ± SD and were plotted by GraphPad Prism 6 software. Statistical analysis was performed using GraphPad Prism 6 software. One-way analysis of variance (ANOVA) or the independent sample *t*-test was conducted to determine the significance between groups. A value with *P* < 0.05 was considered statistical significance.

## 3. Results

### 3.1. Berberine Suppressed the Proliferation of MGC803 Cells

**Figure 1:**
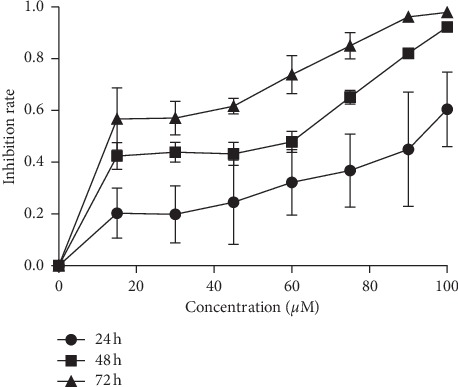
Berberine suppressed the proliferation of MGC803 cells. After treatment with berberine for 24 h, 48 h, and 72 h, the cell growth was determined by MTT assays. Berberine inhibited the growth of MGC803 cells in a time-dependent manner (24 h, 48 h, and 72 h) and in a dose-dependent manner (0–100 *μ*M).

To evaluate the bioactivity of berberine, we performed MTT assays to test the growth inhibition effect of berberine on MGC803 cells. As we can see from Figure 1, berberine significantly retarded the proliferation of MGC803 cells in a time-dependent manner (24 h, 48 h, and 72 h). Besides, Figure 1 also demonstrates that the higher the doses of berberine were, the stronger the inhibition effects were. Berberine inhibited the proliferation of MGC803 cells in a dose-dependent manner (0–100 *μ*M). Therefore, berberine displayed antigrowth properties against gastric cancer MGC803 cells. Considering the cytotoxicity of berberine and given that a certain number of MGC803 cells should be maintained for further experiments, half-maximal inhibitory concentration (IC50, 40 *μ*M) and 1/2 IC50 (20 *μ*M) and 1/4 IC50 (10 *μ*M) of BBR on MGC803 cells at 24 h was chosen for the following experiments.

### 3.2. Berberine Induced Apoptosis of MGC803 Cells

**Figure 2:**
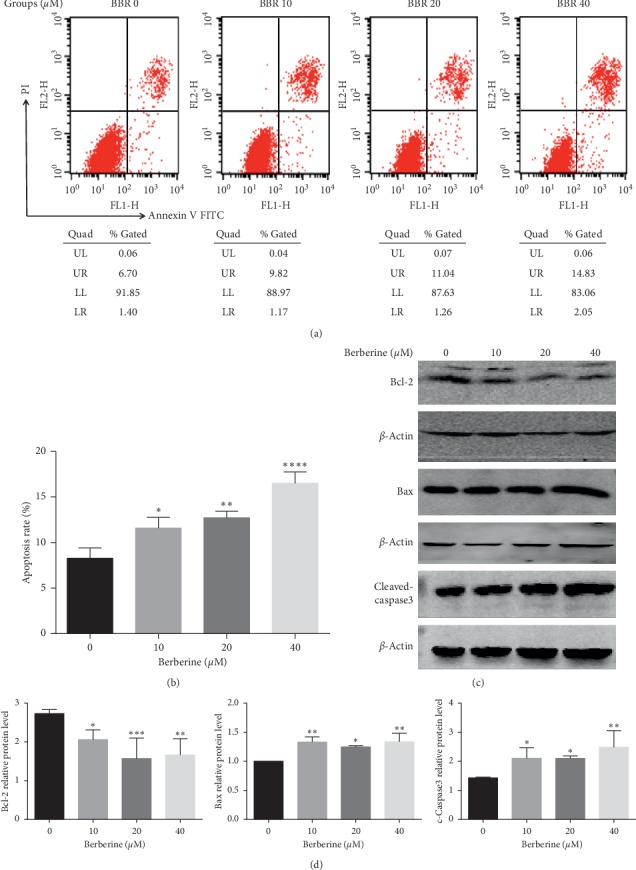
Berberine induced apoptosis of MGC803 cells. (a) Berberine treatment at 10, 20, and 40 *μ*m for 24 h promoted the apoptosis of MGC803 cells, as detected by flow cytometry and statistically analyzed in (b). (c) Berberine treatment at 10, 20, and 40 *μ*m for 24 h downregulated the protein expression of antiapoptosis protein Bcl-2 and promoted the protein expression of proapoptosis protein Bax and activated the expression of caspase3 protein. (d) The protein expression of apoptosis-related proteins of MGC803 cells after berberine treatment was quantified. ^*∗∗∗∗*^*P* < 0.0001, ^*∗∗∗*^*P* < 0.001, ^*∗∗*^*P* < 0.01, and ^*∗*^*P* < 0.05 vs controls.

Apoptosis, which maintains homeostasis through programmed cell death controlled by genes, has long been considered as the biggest challenge in the onset of cancer since it was first proposed by Kerr et al. in 1972 [16]. To explore the mechanisms behind the growth inhibition effects of berberine on MGC803 cells, we conducted the apoptosis assays on MGC803 cells which were treated with or without berberine. We took advantage of Annexin V-FITC/PI apoptosis detection kit to test the apoptotic rate of MGC803 cells induced by berberine. As it is seen in Figures 2(a) and 2(b), berberine induced apoptosis of MGC803 cells and increased the number of apoptotic cells. Besides, treatment with berberine with a dose of 40 *μ*mol/L exerted greater apoptotic effects to MGC803 cells when compared to the other groups, so the higher the concentration of berberine was, the greater the apoptotic effects were. Moreover, we also detected the changes in the protein expression level of apoptosis-related proteins. As it is demonstrated in Figures 2(c) and 2(d), berberine decreased the protein level of antiapoptosis protein Bcl-2 while increased the expression level of proapoptotic protein Bax and activated the caspase3 protein. Thus, berberine inhibited cell survival by promoting the mitochondrial apoptosis pathways.

### 3.3. Berberine Altered the Lipid Contents and Key Lipid Metabolic Enzymes in MGC803 Cells

**Figure 3:**
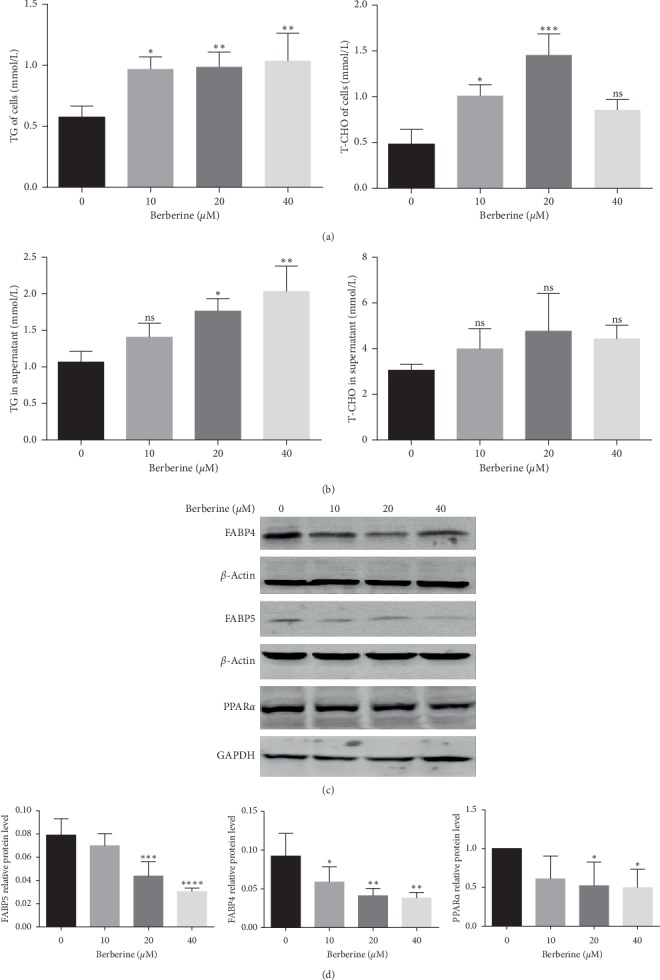
Berberine altered the lipid contents and key lipid metabolic enzymes of MGC803 cells. (a) Berberine treatment at 10, 20, and 40 *μ*M for 24 h increased the level of TG of MGC803 cells. Berberine treatment at 10 and 20 *μ*M for 24 h increased the level of T-CHO while 40 *μ*M had no effects on the level of T-CHO of MGC803 cells. (b) Berberine treatment at 10, 20 and 40 *μ*M for 24 h increased the level of TG while had no effects on the level of T-CHO in the supernatant of MGC803 cells. (c) Berberine treatment at 20 and 40 *μ*M for 24 h suppressed the protein expression of key lipid metabolic enzymes FABP4, FABP5, and PPAR*α* in MGC803 cells. (d) The quantification of protein expression of key lipid metabolic enzymes FABP4, FABP5, and PPAR*α* in MGC803 cells. ^*∗∗∗∗*^*P* < 0.0001, ^*∗∗∗*^*P* < 0.001, ^*∗∗*^*P* < 0.01, ^*∗*^*P* < 0.05, and ns means no statistical significance.

It was pointed out that except for acting as infrastructural elements, lipids, and their metabolism exert key points of control over the Bcl-2 family-regulated mitochondrial apoptotic process [17], which suggested a link between lipids and apoptosis. Recent experiments also declared that berberine regulated lipid metabolism in many diseases such as type 2 diabetes and hepatoma carcinoma cells [18, 19]. Therefore, to investigate the tentative mechanism of how berberine regulated gastric cancer cells, the lipid contents and key lipid metabolic enzymes of MGC803 cells with or without berberine treatment were tested. It was concluded from Figures 3(a) and 3(b) that berberine elevated the contents of triglyceride in the supernatant as well as in MGC803 cells. And these increasing effects were enhanced with higher doses of berberine. Besides, we also detected the level of total cholesterol of MGC803 cells and their supernatant. However, the results in Figures 3(a) and 3(b) show that berberine with a concentration of 10 *μ*mol/L or 20 *μ*mol/L increased the level of total cholesterol of MGC803 cells while berberine with a dose of 40 *μ*mol/L exerted no effects to MGC803 cells. And the level of total cholesterol in the supernatant of MGC803 cells was displayed with no significance after berberine treatment. These might also demonstrate the antitumor effects of berberine, given that route-specific outside-in delivery of cholesterol promotes GC progression [20]. Given that fatty acids of most cells were stored in triglyceride (TG) in cytosolic lipid droplets [21], these results might also suggest that berberine decreased the exogenous fatty acid uptake and lowered the usage of endogenous fatty acid-triglyceride.

Evidence showed that fatty acid uptake across the plasma membrane occurred partially via a protein-mediated process involving several fatty acid-binding proteins (FABPs) known as fatty acid transporters [22, 23]. FABPs are members of the superfamily of lipid-binding proteins, which are responsible for the regulation of fatty acid uptake and intracellular transport [8]. So, we tested the protein level of fatty acid-binding protein component FABP4 and FABP5. It is found in Figures 3(c) and 3(d) that berberine decreased the protein level of both FABP4 and FABP5 of MGC803 cells.

Furthermore, we also determined the expression level of the peroxisome proliferator-activated receptor (PPAR) component PPAR*α*, which channels excess FAs into the FA oxidation pathway, thus playing a vital role in lipid metabolism [24, 25]. Increased PPAR*α* expression could stimulate lipolysis, which in turn reduces lipid deposition [26]. Here as it is shown in Figures 3(c) and 3(d), the protein level of PPAR*α* was suppressed by berberine in MGC803 cells, which might account for the elevated level of fatty acids of MGC803 cells caused by berberine treatment. On the whole, berberine regulated the fatty acid metabolism of MGC803 gastric cancer cells.

### 3.4. The FABP Inhibitor, BMS309403, Could Partially Recapitulate the Effects of Berberine on MGC803 Cells

Emerging evidence suggested that FABPs were associated with the development and progression of cancers [9–11]. A FABP inhibitor, BMS309403, was used to investigate if it could generate a similar effect to that seen in the berberine-treated cells. MGC803 cells were pretreated with a dose of 20 *μ*mol/L BMS309403 for 10 minutes. And then its supernatant was replaced with the complete medium (the BMS group) or 20 *μ*mol/L berberine (the BB group) for 24 h.

**Figure 4:**
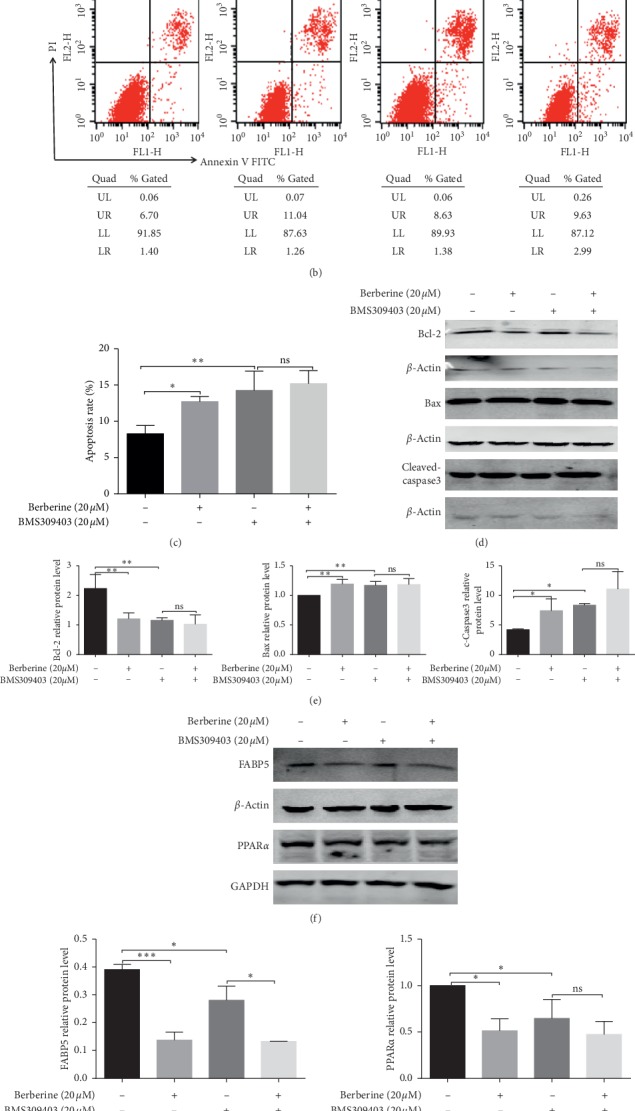
The FABP inhibitor, BMS309403, could partially recapitulate the effects of berberine on MGC803 cells. (a) BMS309403 suppressed the proliferation of MGC803 cells. (b) BMS309403 induced the apoptosis of MGC803 cells, and they were statistically analyzed in (c). (d) BMS309403 treatment downregulated the protein expression of antiapoptosis protein Bcl-2 and promoted the protein expression of proapoptosis protein Bax and activated the expression of caspase3 protein, and these apoptosis-related proteins were quantified in (e). (f) BMS309403 inhibited the protein expression of key lipid metabolic enzymes FABP5 and PPAR*α*. (g) The quantification of the protein expression of key lipid metabolic enzymes FABP5 and PPAR*α* in MGC803 cells after BMS309403 treatment. (h) BMS309403 elevated the cellular contents and supernatant contents of the triglyceride of MGC803 cells. ^*∗∗∗*^*P* < 0.001, ^*∗∗*^*P* < 0.01, ^*∗*^*P* < 0.05, and ns means no statistical significance.

MTT assays were conducted to measure the antiproliferation effects of BMS309403 on MGC803 cells. The results showed that BMS309403 significantly inhibited the growth of MGC803 cells (*P* < 0.05) (Figure 4(a)). Moreover, treatment with BMS309403 plus berberine exerted greater growth inhibition effects than treating with BMS309403 alone (Figure 4(a)), which might lead to the probability that berberine exerted antiproliferation effects partially via inhibiting the FABP pathway.

Besides, to further investigate whether the FABP pathway involved in fatty acid metabolism was responsible for the apoptosis-induced effect of berberine on MGC803 cells, we evaluated the apoptosis rate of MGC803 cells after BMS309403 treatment with or without berberine. It was displayed that BMS309403 significantly elevated the apoptotic rate of MGC803 cells (Figures 4(b) and 4(c)). Besides, berberine also enhanced the protein expression of proapoptosis protein Bax, activated the cleaved-caspase3, and meantime lowered the protein level of antiapoptosis protein Bcl-2 (Figures 4(d) and 4(e)). These indicated that the reduction of the FABPs could result in the trigger of the mitochondrial apoptosis pathway of MGC803 cells. Besides, we also detected the apoptosis-induced effects of berberine on MGC803 cells which were pretreated with BMS309403. As it is seen from Figures 4(e) and 4(f), the level of apoptosis-related proteins including the Bcl-2, Bax, and cleaved-caspase 3 between the BMS group and the BB group was found with no significance, indicating that the decrease of the protein level of FABPs was responsible for the berberine-induced apoptosis effects on MGC803 cells.

Furthermore, the triglyceride contents in supernatant and cancer cells were also increased upon BMS309403 treatment in Figure 4(h), suggesting that the reduction of FABPs might result in the accumulation of fatty acids on MGC803 cells and its supernatants. However, the triglyceride content in supernatant and cancer cells between the BMS group and the BB group was displayed with no significance, indicating that the decrease of the level of FABPs was responsible for the berberine-induced accumulation of fatty acids in MGC803 cells and its supernatants.

We also detected the protein level of PPAR*α* of MGC803 cells which were pretreated with BMS309403 followed by replacement treatment with a complete medium or berberine. Results showed that BMS309403 significantly lowered the expression of PPAR*α*, and there displayed no significance between the BMS group and the BB group in terms of the protein level of PPAR*α* (Figures 4(f) and 4(g)), indicating that the decreased expression of FABPs could result in the reduction of PPAR*α* protein and that berberine decreased the expression of PPAR*α*, at least, partially via targeting FABPs. Therefore, consistent with those observed after berberine treatment, BMS309403 also caused the accumulation of fatty acids and the retardation of tumor growth and induced apoptosis of MGC803 cells.

### 3.5. Berberine Inhibited Tumor Growth in the MGC803 Xenograft Models

**Figure 5:**
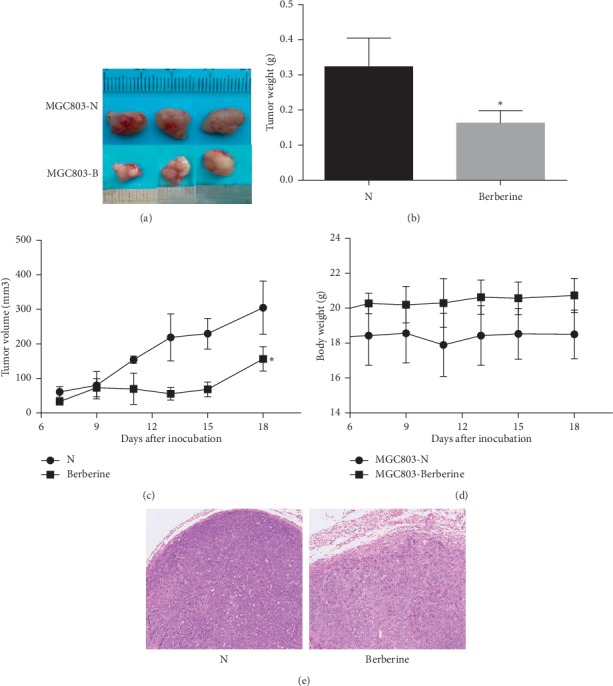
Berberine suppressed tumor growth in the MGC803 xenograft models. (a) Berberine (100 mg/kg/d, intragastric administration) retarded MGC803 xenograft tumor growth. (b) Tumors were removed from the sacrificed mice and weighted. Berberine decreased tumor weight. (c) The tumor volume in the treatment group and the control group. Berberine suppressed tumor growth. (d) Berberine had no effects on the body weights of treated mice. (e) Hematoxylin and eosin (H&E) staining of tumor sections in MGC803 xenografts. ^*∗*^*P* < 0.05.

MGC803 xenograft model was established to explore the potential therapeutic effects of berberine in vivo. As it is shown in Figures 5(a) and 5(b), berberine decreased the tumor size and tumor weight (0.16 ± 0.02 g) (*P* < 0.05) in the MGC803 xenograft models when compared to the control group (0.32 ± 0.04 g). During this experiment, berberine decreased the growth rate of tumors in MGC803 xenografts (Figure 5(c)). Meantime, hematoxylin and eosin (H&E) staining of tumor sections in MGC803 xenografts was performed, and the results displayed that tumors treated with berberine were demonstrated with enlarged intercellular spaces and decreased cell density (Figure 5(e)). Moreover, during this experiment, there existed no significance in terms of weight of mice between the berberine group and the N group (Figure 5(d)), indicating that berberine exerted no side effects on the weight of mice in this experiment. Our research coordinated with other research studies that berberine induced 46.58% inhibition of tumor compared to the control group, showing that berberine significantly reduced the GC tumor incidence and berberine-treated mice displayed significant tumor growth retardation compared with the control group [27].

### 3.6. Berberine Induced Tumor Apoptosis in the MGC803 Xenograft Models

**Figure 6:**
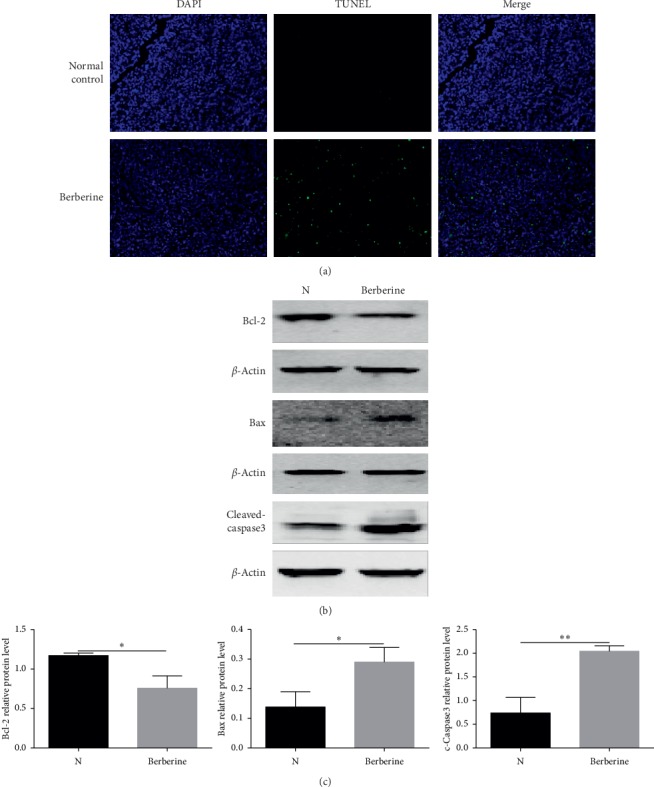
Berberine induced apoptosis of tumors in the MGC803 xenograft models. (a) Berberine treatment induced apoptosis of MGC803 xenograft tumors as detected by TUNEL assays. (b) Berberine downregulated the protein expression of antiapoptosis protein Bcl-2, promoted the protein expression of proapoptosis protein Bax, and activated the expression of caspase3 protein of MGC803 xenograft tumors. (c) The protein expression of apoptosis-related proteins of tumors in MGC803 xenograft models was quantified. ^*∗∗∗∗*^*P* < 0.0001, ^*∗∗∗*^*P* < 0.001, ^*∗∗*^*P* < 0.01, and ^*∗*^*P* < 0.05.

TUNEL assays were also conducted to detect the apoptosis effects induced by berberine in tumor tissues of MGC803 xenografts. As it is displayed in Figure 6(a), treatment with 100 mg/kg/d berberine could lead to a greater apoptosis rate of tumor tissues when compared to the control groups. Besides, we also test the protein level of apoptosis-related proteins of tumor tissues, making use of western blot assays. It was showed that berberine elevated the protein expression level of proapoptotic protein Bax while lowered the level of antiapoptosis protein Bcl-2 when comparing to the control group (Figures 6(b) and 6(c)). Meantime, the protein level of the activated caspase3 protein was also detected and was demonstrated with an increase in tumor tissues of mice which were treated with berberine (Figures 6(b) and 6(c)). So, it could be concluded that berberine induced apoptosis of tumors in the MGC803 xenograft models.

### 3.7. Berberine Altered the Lipid Contents and Key Lipid Metabolic Enzymes in the MGC803 Xenograft Models

**Figure 7:**
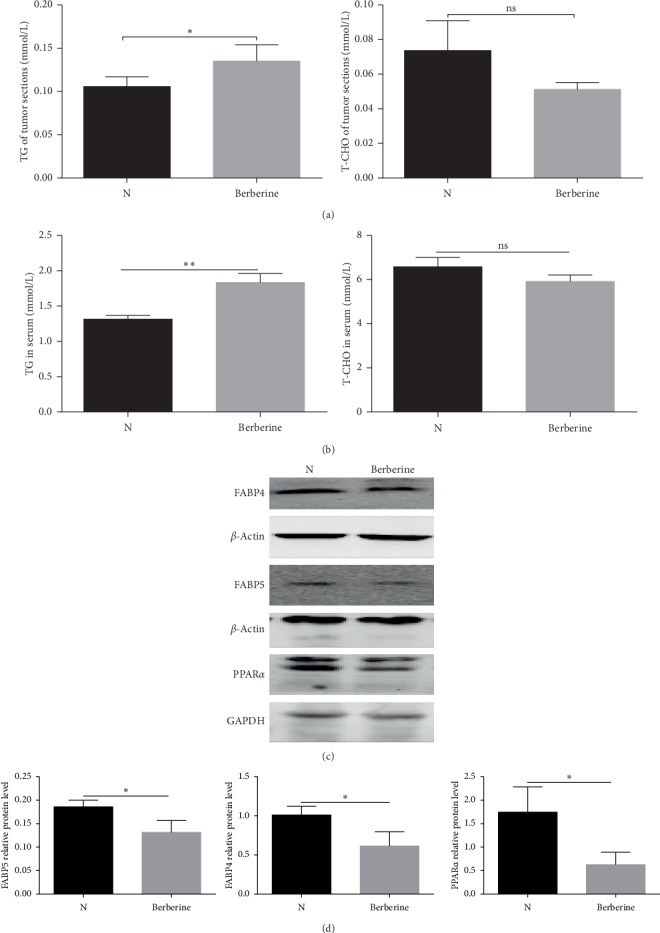
Berberine altered the lipid contents and key lipid metabolic enzymes in the MGC803 xenograft models. (a) Berberine treatment increased the level of TG of MGC803 xenograft tumors. Berberine had no effects on the level of T-CHO of MGC803 xenograft tumors. (b) Berberine treatment elevated the level of TG in the serum of MGC803 xenografts. Berberine had no effects on the level of T-CHO in the serum of MGC803 xenografts. (c) Berberine suppressed the protein expression of key lipid metabolic enzymes FABP4, FABP5, and PPAR*α* of MGC803 xenograft tumors. (d) The quantification of key lipid metabolic enzymes FABP4, FABP5, and PPAR*α* of tumor tissues from MGC803 xenografts. ^*∗∗*^*P* < 0.01, ^*∗*^*P* < 0.05, and ns means no statistical significance.

We also tested the lipid contents of tumors and the lipid contents in serum in MGC803 xenografts. As it is presented in Figure 7(a), the level of the triglyceride of tumor tissues was much higher in the berberine group than that in the control group. Treatment with berberine was also found to elevate the level of triglyceride in the serum of MGC803 xenografts in Figure 7(b). However, the level of total cholesterol of tumors and the total cholesterol in serum was displayed with no significance between the berberine group and the control group (Figures 7(a) and 7(b)). Furthermore, the key lipid metabolic enzymes involved in the regulation of fatty acid metabolism were also detected (Figures 7(c) and 7(d)). It was found that the protein expression level of PPAR*α* of tumor sections was retarded by berberine in the MGC803 xenograft models. Besides, it was also demonstrated that the expression level of both FABP4 and FABP5 was downregulated by treatment with berberine in tumor tissues of MGC803 xenografts. Therefore, it was concluded that berberine regulated fatty acid metabolism in the MGC803 xenograft models.

## 4. Discussion

According to the traditional Chinese medicine theories, Coptidis Rhizoma is routinely used to remove damp-heat and fire which are regarded as external pathogenic factors that cause diseases. Although the pathological concept of cancer first appeared in modern medical theories, it is still easy to discover some descriptions of cancer-like symptoms in ancient medical records in China. In the ancient Chinese medical monographs “ZhongZangJing,” cancer was described as “Yong, Yang, Chuang, and Zhong,” which are caused by intracorporal retention of various pathogens including heat and dampness [28]. Therefore, the use of clearing heat and dampness of the traditional Chinese medicine Coptidis Rhizoma and its abstract berberine indicates its potential therapeutic use for treating cancer.

Accumulating experiments have contributed to the antitumor properties of Coptidis Rhizoma extract and its active component berberine. Berberine could downregulate IL-8 expression through inhibition of the EGFR/MEK/ERK pathway to suppress cell invasiveness and growth in triple-negative breast cancer cells [29]. Berberine also could inhibit angiogenesis in glioblastoma xenografts by targeting the VEGFR2/ERK pathway [30]. Besides, berberine was also illustrated to promoted apoptosis of colorectal cancer via regulation of the long noncoding RNA (lncRNA) cancer susceptibility candidate 2 (CASC2)/AU-Binding Factor 1 (AUF1)/B-Cell CLL/Lymphoma 2 (Bcl-2) Axis [31]. Here, in this study, we observed that berberine retarded the growth of human gastric cancer cells and induced apoptosis through the mitochondrial apoptosis pathway. These berberine-induced apoptotic effects likely arose, in part, from the effects of berberine on cellular lipid homeostasis [32].

Lipids which mainly include fatty acids and cholesterol are vital sources of energy metabolism in the human body [33]. Studies suggested that to meet the needs of biosynthesis during high levels of proliferation, the reprogramming of fatty acid (FA) metabolism becomes essential in cancer cells [34]. The abnormal fatty acid metabolism affects numerous vital cellular processes including cell growth, proliferation, and survival [17, 35], thus playing a crucial role in cancer development and progression [36, 37]. And it has been proposed that increased storage of fatty acids in neutral lipids (such as TGs) could lead to a reduction in available fatty acids, which was used for membrane building blocks or signaling lipids and inhibited cellular proliferation [23, 36], just the same with that observed in MGC803 cells after berberine treatment. We found that the berberine treatment of MGC803 cells led to the accumulation of fatty acid-triglyceride. Mounting evidence highlights the membrane as an equally important factor in the successful induction of the death response, though the mitochondrial apoptotic process heavily relies on its protein components [17, 23]. Unger et al. found that excess free fatty acids in the cytoplasm are harmful to cells: they can disrupt mitochondrial membrane integrity [38]. In our experiments, we found that treatment with berberine induced an accumulation of fatty acids and activated Bcl-2 family-regulated mitochondrial apoptotic process in MGC803 cells. These findings suggested that the accumulation of fatty acids was responsible for the activation of the mitochondrial apoptotic process.

Cancer cells are often in a metabolically challenging atmosphere with scarce availability of oxygen and nutrients, and such metabolic stress can lead to changes in the balance between the endogenous synthesis and exogenous uptake of fatty acids, which are needed for membrane biogenesis, energy production, and protein modification of cells [24]. It was pointed out that hypoxic cancer cells might turn to fatty acid uptake pathways to compensate for the repressed glucose-based de novo fatty acid synthesis [39]. The long-chain fatty acid uptake across the plasma membrane occurs mainly via several fatty acid-binding proteins (FABPs), which positively correlates with rates of fatty acid transport [23]. Nieman et al. showed that adipocytes surrounding tumor cells provide energy through supplying fatty acids to cancer cells in the FABP-dependent mechanism [40]. FABPs are cytoplasmic proteins, which could bind and transfer long-chain fatty acids to different intracellular destinations [41]. FABPs are recognized as a protective sink for long-chain fatty acids defending cells from injuries by excessive lipid accumulation [23]. Here, we found that the protein level of FABPs was downregulated and the accumulation of triglyceride was also induced by berberine in MGC803 cells and in MGC803 xenograft models. These results indicated that the reduction of FABPs resulted from berberine treatment could lead to the accumulation of fatty acids.

Recently, more and more researches have focused on the role of FABPs in the development and progression of a variety of cancers. The epidermal-type fatty acid-binding protein-5 (FABP-5) gene acts as a key molecule in the development and progression of a variety of tumors like breast cancer [42] and hepatocellular carcinoma [43]. Studies have shown that the upregulation of FABP5 stimulates fatty acid transport, which finally induces cell growth and survival [44]. It was also presented that the silence of the FABP5 gene might attenuate the invasiveness of gastric cancer cells, detain cell proliferation, and arrest the cell cycle in the G0/G1 phase, leading to a significant increase in apoptosis [45]. BMS309403 is a well-designed inhibitor of FABPs (FABP3-5, and −7), which interacts with the fatty acid-binding pocket to inhibit the binding of fatty acids [23, 46]. In our studies, the BMS309403 phenocopy was identical to the effects of berberine on lipid metabolism and cell apoptosis in MGC803 cells. We discovered that the FABP inhibitor BMS309403 could result in the accumulation of triglyceride, induce apoptosis, and suppress the growth of MGC803 cells. Our results correspond with the results that exogenous FABP reduces active caspase 3 expressions in HepG2 cells, which effect is reversed in the presence of BSM309403 [47].

In summary, we found that treatment with berberine inhibited gastric cancer growth in vitro and in vivo by suppressing the expression of FABPs, which led to the accumulation of fatty acids and apoptosis of gastric cancer cells. Our experiments provided new insight into the potential pharmacological application of berberine as an antitumor agent and also indicated new clues for biomarkers or therapeutic targets of lipid metabolism in tumors. Our studies also enriched the metabolic regulatory mechanism of berberine against gastric cancer.

## 5. Conclusion

Berberine, a natural isoquinoline alkaloid isolated from herbal plants such as Coptis, exerted anticancer effects both in vitro and in vivo by inducing apoptosis, due to the accumulation of fatty acids and the reduced expression of FABPs, suggesting that berberine was a promising novel anticancer agent.

## Data Availability

The data used to support the findings of this study are available from the corresponding author upon request.

## Supplementary Materials

Supplementary Materials Figure 2(c): all immunoblots of apoptosis-related proteins Bcl-2, Bax, and cleaved-caspase3 of MGC803 cells after berberine treatment. Figure 3(c): all immunoblots of key lipid metabolic enzymes FABP4, FABP5, and PPAR*α* in MGC803 cells after berberine treatment. Figure 4(d): All immunoblots of Bcl-2, Bax, and cleaved-caspase3 of MGC803 cells after BMS309403 treatment. Figure 4(f): all immunoblots of key lipid metabolic enzymes FABP5 and PPAR*α* of MGC803 cells after BMS309403 treatment. Figure 6(b): all immunoblots of apoptosis-related proteins Bcl-2, Bax, and cleaved-caspase3 of MGC803 xenograft tumors. Figure 7(c): all immunoblots of key lipid metabolic enzymes FABP4, FABP5, and PPAR*α* of MGC803 xenograft tumors.
