# Supplementary material for: Berberine Suppressed Tumor Growth through Regulating Fatty Acid Metabolism and Triggering Cell Apoptosis via Targeting FABPs
Source: Evid Based Complement Alternat Med. 2020 Apr 8;2020:6195050. doi: 10.1155/2020/6195050 (PMC7168748; doi:10.1155/2020/6195050)
Supplement: Supplementary Materials — Figure 2(c): all immunoblots of apoptosis-related proteins Bcl-2, Bax, and cleaved-caspase3 of MGC803 cells after berberine treatment. Figure 3(c): all immunoblots of key lipid metabolic enzymes FABP4, FABP5, and PPARα in MGC803 cells after berberine treatment. Figure 4(d): All immunoblots of Bcl-2, Bax, and cleaved-caspase3 of MGC803 cells after BMS309403 treatment. Figure 4(f): all immunoblots of key lipid metabolic enzymes FABP5 and PPARα of MGC803 cells after BMS309403 treatment. Figure 6(b): all immunoblots of apoptosis-related proteins Bcl-2, Bax, and cleaved-caspase3 of MGC803 xenograft tumors. Figure 7(c): all immunoblots of key lipid metabolic enzymes FABP4, FABP5, and PPARα of MGC803 xenograft tumors. [file 6195050.f1.pdf]

Western Blot:

Figure 2c

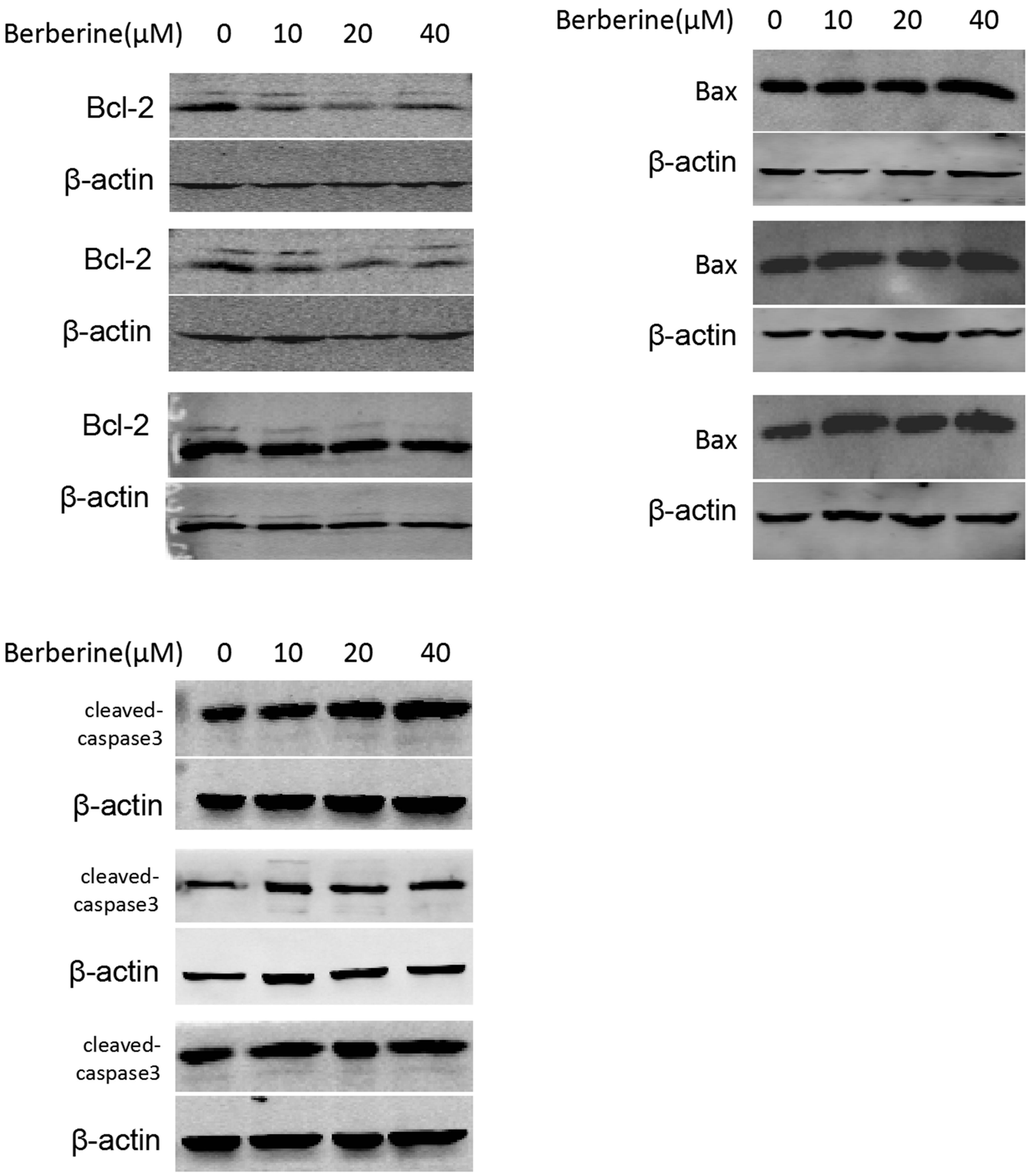

Figure 2(c). All immunoblots of protein-related proteins Bcl-2, Bax and cleaved-caspase3 of MGC803 cells after berberine treatment. Berberine treatment at 10, 20 and 40μm for 24h down-regulated the protein expression of anti-apoptosis protein Bcl-2 and promoted the protein expression of pro-apoptosis protein Bax and activated the expression of caspase3 protein.

Figure 3c

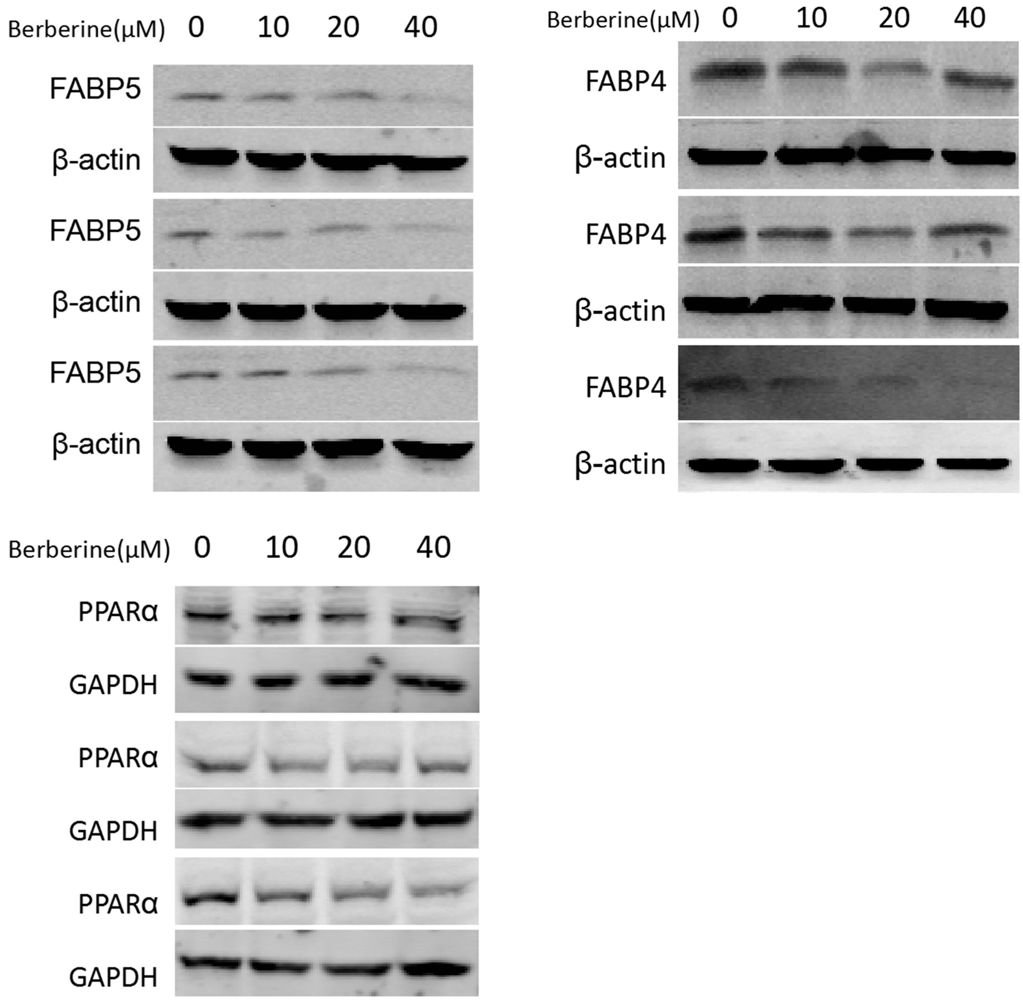

Figure 3(c). All immunoblots of key lipid metabolic enzymes FABP4, FABP5, and PPARα in MGC803 cells after berberine treatment. Berberine treatment at 20 and 40μM for 24h suppressed the protein expression of key lipid metabolic enzymes FABP4, FABP5, and PPARα in MGC803 cells.

Figure 4d

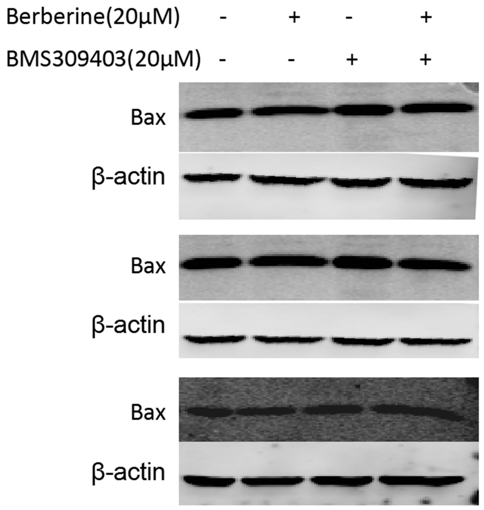

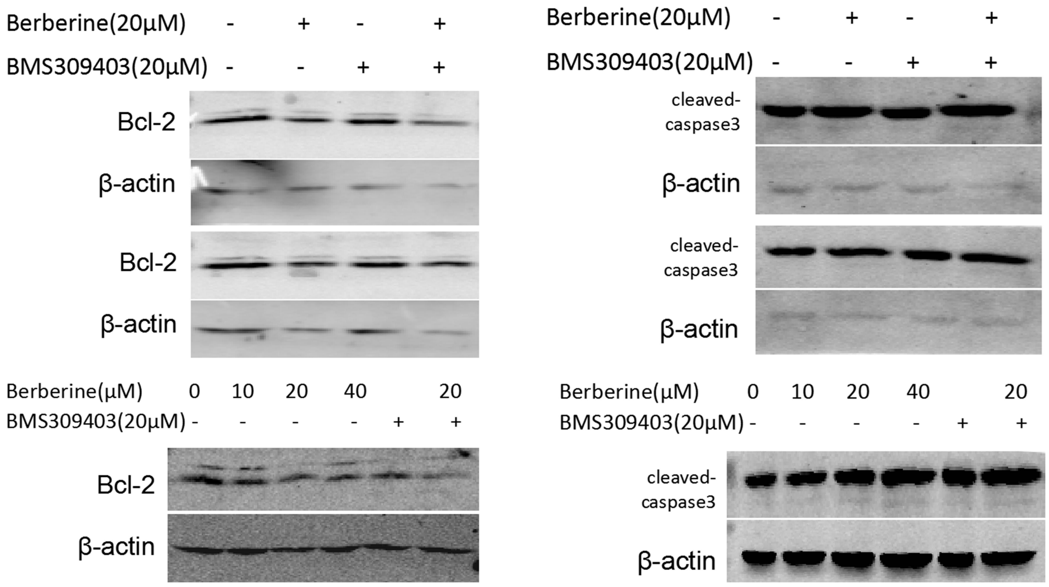

Figure 4(d). All immunoblots of Bcl-2, Bax and cleaved-caspase3 of MGC803 cells after BMS309403 treatment. BMS309403 treatment downregulated the protein expression of anti-apoptosis protein Bcl-2 and promoted the protein expression of pro-apoptosis protein Bax and activated the expression of caspase3 protein.

Figure 4f

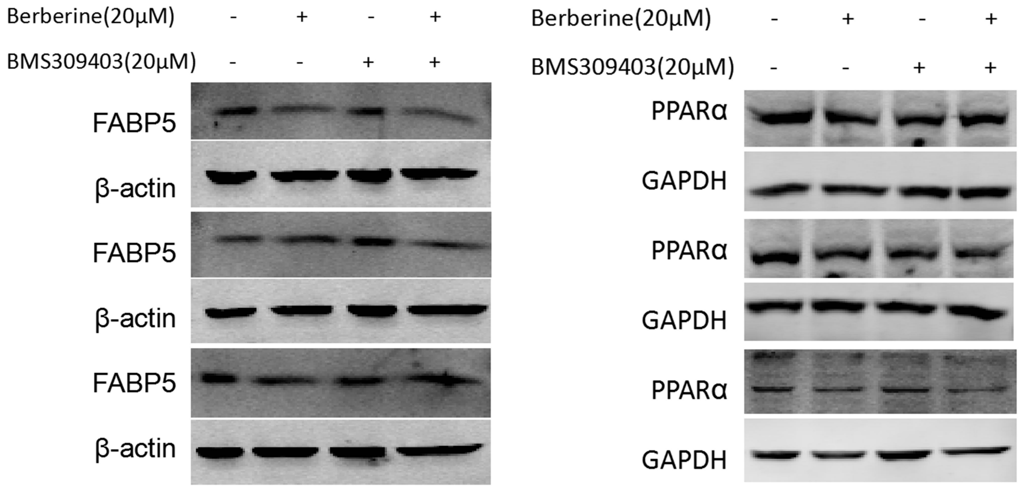

Figure 4(f). All immunoblots of key lipid metabolic enzymes FABP5 and PPARα of MGC803 cells after BMS309403 treatment. BMS309403 inhibited the protein expression of key lipid metabolic enzymes FABP5 and PPARα.

Figure 6b

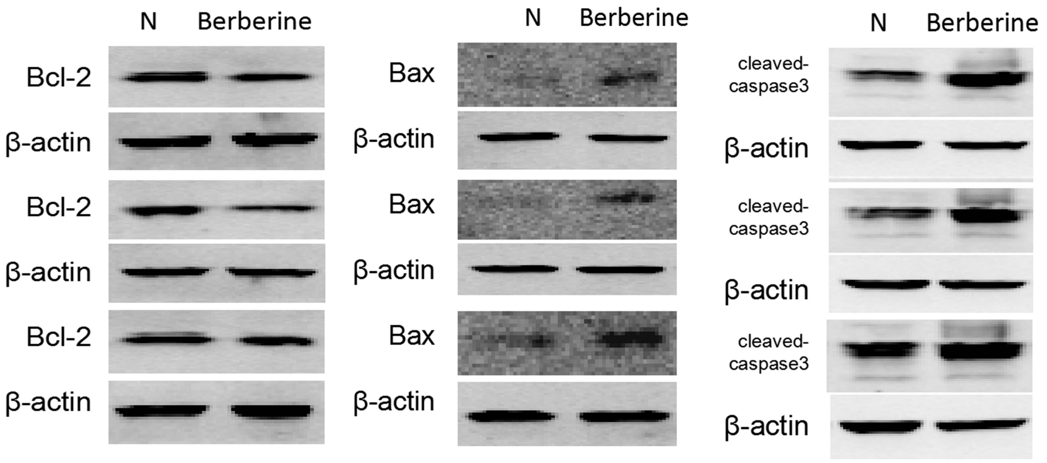

Figure 6(b). All immunoblots of apoptosis-related proteins Bcl-2, Bax and cleaved-caspase3 of MGC803 xenograft tumors. Berberine downregulated the protein expression of anti-apoptosis protein Bcl-2 and promoted the protein expression of pro-apoptosis protein Bax and activated the expression of caspase3 protein of MGC803 xenograft tumors.

Figure 7c

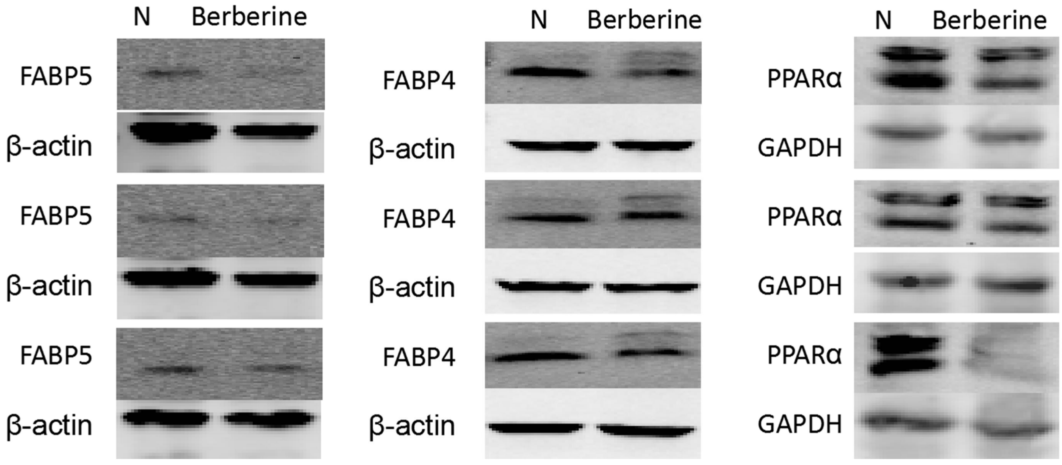

Figure 7(c). All immunoblots of key lipid metabolic enzymes FABP4, FABP5, and PPARα of MGC803 xenograft tumors. Berberine suppressed the protein expression of key lipid metabolic enzymes FABP4, FABP5, and PPARα of MGC803 xenograft tumors.
